# Implementación de la farmacogenética en atención primaria

**DOI:** 10.1016/j.aprim.2024.102881

**Published:** 2024-02-19

**Authors:** Ismael Ejarque Doménech, Antonio Souviron Rodríguez, María José Herrero Cervera, Adrián Llerena

**Affiliations:** a Centro de Salud de Almácera, Almácera, Valencia, España; b Hospital Vithas Aguas Vivas, Carcaixent, Valencia, España; c Centro de Salud El Palo, Málaga, España; d Departamento de Farmacología, Facultad de Medicina, Universidad de Málaga, Málaga, España; e Departamento de Farmacología, Universidad de Valencia, Valencia, España; f Unidad de Farmacogenética y Terapia Génica, Instituto de Investigación Sanitaria La Fe, Valencia, España; g Unidad de Farmacogenética y Medicina Personalizada, Hospital Universitario de Badajoz, Badajoz, España; h Instituto Universitario de Investigación Biosanitaria de Extremadura (INUBE), Badajoz, España; i Facultad de Medicina, Universidad de Extremadura, Badajoz, España

*Sr. Editor,*

El pasado 7 de junio de 2023 la Comisión de Prestaciones, Aseguramiento y Financiación acordó la propuesta de pruebas genéticas/genómicas en el Sistema Nacional de Salud (SNS) que se presenta el 23 de enero de 2024. Dicha propuesta incluye la realización de pruebas farmacogenéticas/farmacogenómicas, lo cual, sin duda, es imprescindible para una estrategia nacional de Medicina Personalizada de Precisión (MPP). Pensamos que la atención primaria (AP) debe jugar un papel central en dicha implementación y no quedar al margen de algo tan importante que va a cambiar la manera en que se prescriben fármacos en medicina. No se puede seguir con el antiguo método de prueba-error. En su lugar, debemos emplear biomarcadores farmacogenómicos siempre que esto sea posible y para ello deben estar disponibles para el médico de AP en su trabajo habitual, y este debe saber interpretarlos correctamente.

El desarrollo de la farmacogenética en el contexto de la MPP está cambiando el paradigma: seleccionar el fármaco y las dosis más adecuados en la mejor combinación con otros; todo ello teniendo en cuenta el perfil genético del paciente. Es decir, hay que prescribir fármacos seleccionándolos, recomendando dosificación y comedicación según las diferentes variantes que cada paciente presentará en los genes relacionados con el recorrido del fármaco por nuestro organismo. Por ejemplo, para el médico de AP, conocer cuáles de sus pacientes tienen mayor probabilidad de presentar mialgias o incluso rabdomiólisis con el empleo de simvastatina o atorvastatina, mediante el análisis de ciertas variantes en el gen *SLCO1B1*, le permitirá ajustar el tratamiento de la forma más conveniente. Esto constará en la historia clínica, tanto de AP como hospitalaria, para sucesivas consultas. Es algo sin duda fácil y económico para la importancia que supone en el tratamiento de los pacientes y en la sostenibilidad del SNS.

La AP es un elemento determinante de los sistemas sanitarios del mundo y específicamente de Europa. El médico de familia y el pediatra de AP son responsables del cuidado continuo del paciente y en la mayoría de los casos de la coprescripción de fármacos. La MPP puede determinar el fármaco idóneo respecto de una diana, algo que se suele producir en las consultas especializadas, pero son el médico de familia y el pediatra de AP los que determinan el tratamiento concomitante que puede ser determinante de la salud del paciente.

En Extremadura lleva ya un tiempo el Proyecto de Implementación de la Farmacogenética en el Servicio Extremeño de Salud: MedeA (www.proyectomedea.es), sobre farmacogenética y MPP, en el que la cohorte de AP Lusitania tiene una relevancia destacada.

El 23 de junio de 2023 el Ministerio de Sanidad aprobó la cartera de servicios genómicos para nuestro SNS, decisión que fue posteriormente ratificada por el Consejo Interterritorial del SNS^1^. En la tabla 1 se muestran las pruebas farmacogenéticas que se incluyen en dicha cartera de servicios genómicos.

**Tabla 1:** Fármacos con prueba farmacogenética que se incluyen en la cartera de servicios genómicos del Sistema Nacional de Salud

Medicamento	Gen	Medicamento	Gen	Medicamento	Gen
Abacavir	*HLA-B*	Fenitoína	*HLA-B*	Rasburicasa	*G6PDH*
Alopurinol	*HLA-B*	Fluorouracilo	*DPYD*	Simvastatina	*SLCO1B1*
Atazanavir	*CYP2C19*	Irinotecán	*UGT1A1*	Siponimod	*CYP2C9*
Azatioprina	*NUDT15, TPMT*	Ivacaftor	*CFTR*	Tegafur	*DPYD*
Capecitabina	*DPYD*	Mercaptopurina	*NUDT15, TPMT*	Tetrabenazina	*CYP2D6*
Carbamazepina	*HLA-A, HLA-B*	Omeprazol	*CYP2C19*	Tioguanina	*NUDT15*
Clopidogrel	*CYP2C19*	Oxcarbazepina	*HLA-B*	Voriconazol	*CYP2C19*
Eliglustat	*CYP2D6*	Pimozida	*CYP2D6*		

Consultar Consejo Interterritorial. Sistema Nacional de Salud.^1^ para conocer bajo qué indicaciones específicas se incluye la prueba y cuáles son las variantes genéticas a analizar. También hay otros fármacos de uso secundario.

Mientras cada comunidad autónoma establece el circuito asistencial, serán necesarias sin duda la formación y la actualización del colectivo de médicos de familia y pediatras de AP para poder utilizar esta herramienta en la toma de decisiones para la prescripción. La participación de las organizaciones profesionales y las sociedades científicas es relevante.

Esperemos que en un futuro no muy lejano se disponga de los datos genéticos de la historia clínica electrónica (tanto en AP como hospitalaria) en toda España para pautar el mejor medicamento para el paciente. Queda camino por recorrer, pero España ya es pionera en Europa al contar con pruebas de este tipo en la cartera pública y común de servicios del SNS.

## Conflicto de intereses

Ninguno de los 4 coautores presenta conflicto de intereses.
